# Supplementary material for: Characterization of a Novel RNA Virus Causing Massive Mortality in Yellow Catfish, Pelteobagrus fulvidraco, as an Emerging Genus in Caliciviridae (Picornavirales)
Source: Microbiol Spectr. 2022 Aug 4;10(4):e00624-22. doi: 10.1128/spectrum.00624-22 (PMC9431444; doi:10.1128/spectrum.00624-22)
Supplement: Supplemental file 1 — Supplemental material. Download spectrum.00624-22-s0001.pdf, PDF file, 0.4 MB [file spectrum.00624-22-s0001.pdf]

FIG S1 Fluorescence *in situ* hybridization (FISH) detection of YcCV in different tissues from naturally infected yellow catfish. (A-D) FISH hybridization in YcCV-infected liver, heart, gill, and intestine cells, respectively; Positive signals detected in the liver, heart, and gills (Aa-Ca), respectively; no positive signals were detected in the intestine cells (Da）; The blue signals showing staining of cell nuclei (Ab-Db) (bar = 50 mm).

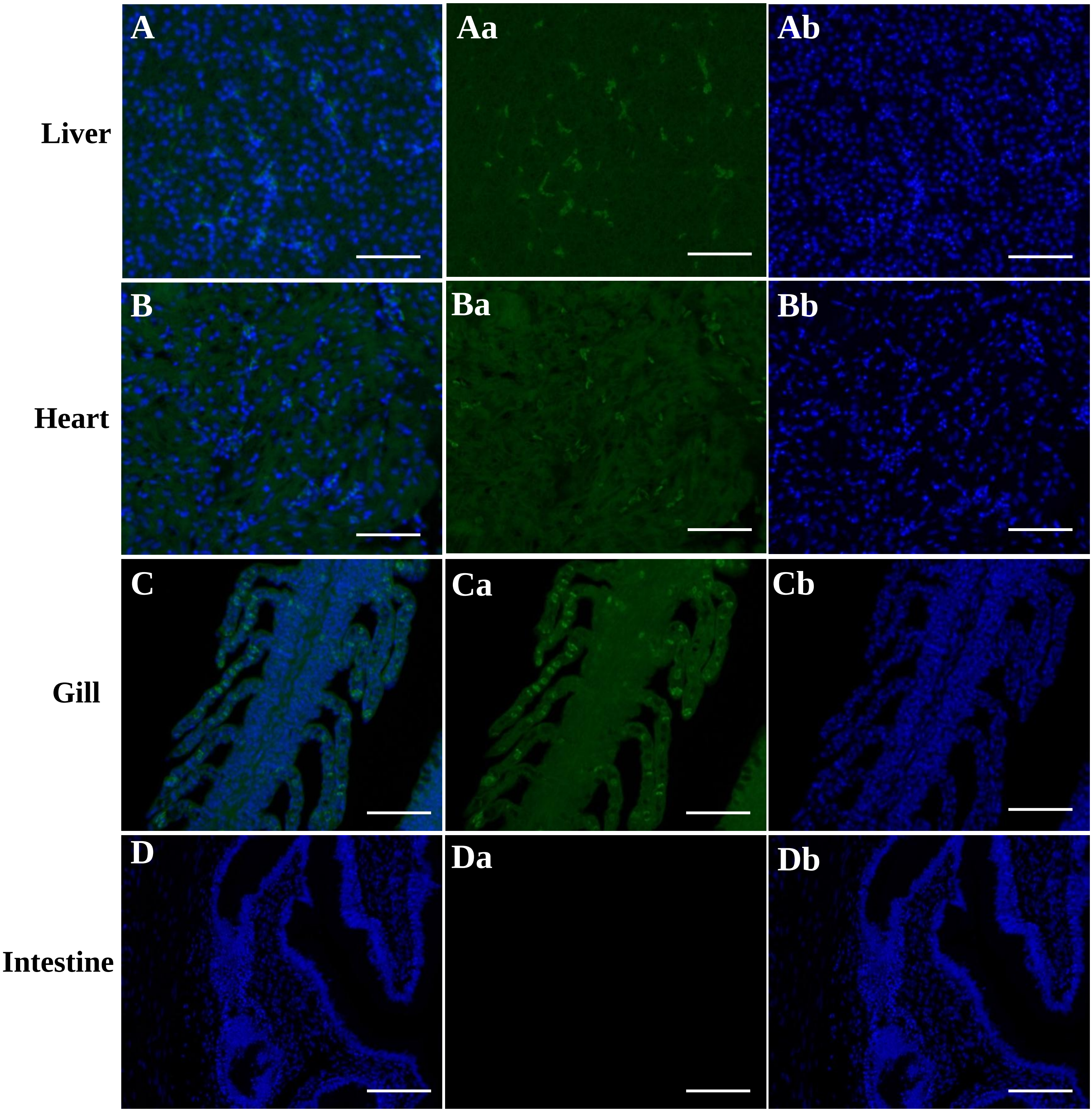
Liver
Heart
Gill
Intestine
A
Aa
Ab
B
Ba
Bb
C
Ca
Cb
D
Da
Db

TABLE S1. Origins of the YcCV detected from different provinces in this study

Sampling location	Abbreviation	Province	Date of collection	Temperature (°C)	Size (cm)
Qianjiang	QJ		25 Apr. 2020	20	9~14
Jingmen	JM		30 Apr. 2020	22	8~15
Honghu	HH		12 May 2020	23	10~13
	JY			24	9~13
Wuhan	WH	Hubei	15 Mar. 2021	20	9~15
Xiantao	XT		2 Apr. 2021	21	8~12
Yichang	YC		10 Apr. 2021	22	10~15
Zhijiang	ZJ		17 Apr. 2021	22	9~12
Jingzhou	JZ		23 Apr 2021	23	10~15
Leshan	LS	Sichuan	9 Apr. 2021	20	10~12
